# A JAK of two trades: beneficial or adverse effects of Janus kinase inhibition for plasma LDL-cholesterol and major adverse cardiovascular events in inflammatory bowel disease patients?

**DOI:** 10.1097/MOL.0000000000001040

**Published:** 2026-04-07

**Authors:** Katherine Muñoz Ayala, Eleonora A.M. Festen, Marit Westerterp

**Affiliations:** a Department of Gastroenterology and Hepatology; b Department of Pediatrics, University of Groningen, University Medical Center Groningen, Groningen, the Netherlands

**Keywords:** cardiovascular disease, inflammatory bowel disease, Janus kinase inhibitors, LDL-cholesterol

## Abstract

**Purpose of review:**

Janus kinase (JAK) inhibitors have emerged as potent anti-inflammatory drugs and are prescribed in rheumatoid arthritis (RA) and inflammatory bowel disease (IBD). However, cardiovascular safety concerns have emerged from RA safety trials, alongside increases in LDL cholesterol (LDL-c). Whether similar lipid changes in IBD translate into clinically meaningful cardiovascular risk remains uncertain. This review focuses on studies and mechanisms increasing LDL-c due to JAK inhibition in IBD.

**Recent findings:**

JAK inhibitors increase plasma LDL-c levels in IBD patients in a dose-dependent manner. While this has generally been attributed to JAK inhibitors decreasing inflammation, inhibition of JAK signaling also decreases hematopoiesis: the differentiation of hematopoietic stem cells into granulocyte/macrophage progenitors, and subsequently neutrophils, monocytes, and macrophages. Hematopoiesis requires LDL-c uptake, and is associated with elevated plasma LDL-c when inhibited. While statins lower LDL-c, the high prevalence of metabolic syndrome-associated type 2 diabetes in IBD patients may prompt the use of alternative lipid-lowering drugs.

**Summary:**

JAK inhibitors are associated with increased LDL-c levels in IBD patients which may be due to suppressed inflammation and decreased hematopoiesis. This review summarizes the evidence on lipid changes and cardiovascular outcomes upon JAK inhibition and discusses approaches for LDL-c management in IBD.

## INTRODUCTION

Over the past decade, Janus kinase (JAK) inhibitors have emerged as potent anti-inflammatory drugs. JAK inhibitors are being actively prescribed for several pro-inflammatory disease phenotypes, including rheumatoid arthritis (RA) and inflammatory bowel disease (IBD) such as ulcerative colitis and Crohn's disease. Unlike antibody therapies that target individual cytokines in IBD, JAK inhibitors modulate the production of several cytokines simultaneously, leading to a broader immunomodulatory effect, resulting in therapeutic benefit for patients who are refractory or intolerant to antibody drugs [1^▪^]. Importantly, JAK inhibitors induce and maintain remission in ulcerative colitis and Crohn's disease patients. Despite their anti-inflammatory benefits, several JAK inhibitors have received a Food and Drug Administration (FDA) warning [2], as they increase the risk of major adverse cardiovascular events (MACE) in RA patients, presumably due to an increase in plasma LDL-cholesterol (LDL-c) levels [3]. While RA is classically associated with increased cardiovascular risk [4,5], and JAK inhibitors may cause MACE in RA patients with an unfavorable cardiovascular profile, it is unclear whether this can be extrapolated to other inflammatory diseases, including IBD.

A recent meta-analysis has shown that JAK inhibitors increase LDL-c in patients with several immune-mediated diseases [6^▪▪^]. However, the analysis primarily involved RA cohorts, and while the pooled results indicated that JAK inhibition significantly increased plasma LDL-c, only one study on IBD, in this case Crohn's disease, was included (Table 1). Dose-stratified analyses revealed that plasma LDL-c changes primarily occurred at higher doses of JAK inhibitors [7]. However, the clinical relevance of this lipid signal is unsettled: evidence linking JAK inhibitor-associated LDL-c changes to cardiovascular outcomes in IBD is limited, and the current caution mainly derives from RA safety data and regulatory risk communications. Recent consensus recommendations emphasize structured risk stratification and monitoring, but they also highlight how many decisions are currently made under uncertainty [8^▪▪^]. This review will focus on mechanisms mediated by JAK inhibition that increase plasma LDL-c levels in IBD, integrating current clinical evidence, and discussing drug-specific approaches to mitigate these lipid changes. 

**Box 1:**
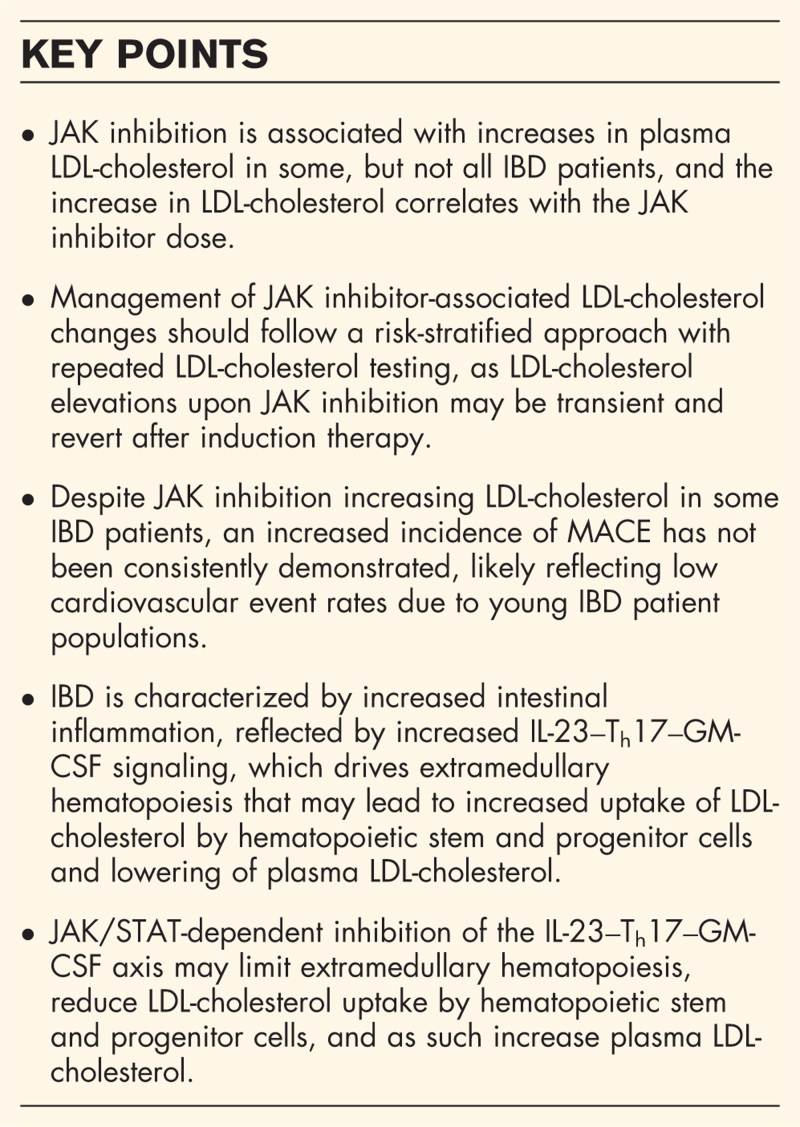
no caption available

## PLASMA LDL-CHOLESTEROL LEVELS IN INFLAMMATORY BOWEL DISEASE AND EFFECTS OF JANUS KINASE INHIBITORS

A recent systematic review and meta-analysis reported that plasma LDL-c levels are significantly lower in IBD patients compared to healthy controls [9]. These data would suggest that IBD patients are at lower cardiovascular risk; however, studies evaluating LDL composition revealed a high plasma level of small dense LDL particles in IBD. Small dense LDL particles are highly pro-inflammatory and increase cardiovascular risk [10,11^▪^]. As a consequence, the term LDL paradox was coined, meaning that LDL-c levels in IBD, and other auto-immune diseases, such as RA, are low, but due to the particle composition, LDL particles are more pro-atherogenic than in healthy controls [10,11^▪^]. Indeed, a recent meta-analysis of 14 studies did show a modest increase in cardiovascular risk (myocardial infarction, ischemic heart disease, cerebrovascular accident, and MACE) in patients with IBD [12^▪▪^].

In addition, even though data on IBD being associated with low LDL-c originated from a meta-analysis of 53 studies [9], the opposite, namely high plasma levels of LDL-c in IBD patients, has also been reported [13,14]. These apparent conflicting results on LDL-c have been reviewed previously and were attributed to the level of inflammation in the intestine, with only very high levels of inflammation associated with low LDL-c [15]. Indeed, the majority of studies show that severe IBD is associated with low plasma LDL-c levels. This has mainly been attributed to severe inflammation triggering the acute phase response in the liver leading to inhibition of cholesterol synthesis in hepatocytes and less production of very-low-density lipoprotein (VLDL)-cholesterol, as such lowering LDL-c in IBD patients [15].

The tight connection between inflammation and LDL-c in IBD patients is further supported by patients with active IBD having lower LDL-c levels than those in remission [9]. Further, clinical trials in IBD have shown that JAK inhibitors which decrease inflammation, induce changes in LDL-c plasma levels in a dose-dependent manner. This occurred regardless of the type of JAK inhibitor used (Table 1). Higher doses during JAK inhibitor induction therapy increased plasma LDL-c levels, while lower doses tended to produce smaller or nonsignificant changes [16]. Studies on whether this translates to long-term effects of JAK inhibitors on plasma LDL-c levels during maintenance therapy have yielded conflicting results. Some studies have shown that LDL-c levels tend to return to baseline after induction therapy and generally remain stable [17,18], whereas others showed LDL-c increases during maintenance therapy [19]. Notably, these observations are based on studies with relatively short follow-up (16–58  weeks) [6^▪▪^,^18^,^20^,^21^], leaving the long-term effects of JAK inhibitors on LDL-c changes uncertain. In addition, effects of the JAK inhibitor tofacitinib on small-dense LDL particles in the context of RA and psoriasis showed either no effect (in psoriasis) [22] or a decrease (in RA) [23]. Its effects on small dense LDL will need to be evaluated in IBD.

Whether JAK inhibition-induced changes in LDL-c levels translate into clinically meaningful effects on MACE in IBD remains unresolved. The relatively young IBD population leads to an under-representation of patients at higher cardiovascular risk in the clinical trials and, consequently a low incidence of MACE, limiting statistical power of the trials, as reported in a recent meta-analysis [12^▪▪^]. Although cardiovascular outcomes have been evaluated in elderly IBD cohorts, these observational studies report low absolute numbers of MACE events and have limited follow-up periods [24,25].

## MECHANISTIC INSIGHTS INTO HOW JANUS KINASE MUTATIONS AND INHIBITORS AFFECT PLASMA LDL-CHOLESTEROL LEVELS

While earlier reviews have attributed the increases in LDL-c upon JAK inhibition to decreases in intestinal inflammation, and mucosal healing [26], additional mechanisms may play a role.

Additional evidence for a link between the JAK pathway and plasma LDL-c levels mainly comes from studies on the *JAK2* p.Val617Phe mutation. The *JAK2* p.Val617Phe mutation is associated with myeloproliferative diseases (MPD) [27]. *JAK2* p.Val617Phe mutation carriers have low LDL-c levels [28]. In a previous review [29], we attributed the low LDL-c levels in *JAK2* p.Val617Phe mutation carriers to increased proliferation of hematopoietic stem cells (HSCs) that are precursors of granulocyte–macrophage progenitors (GMPs), and subsequently differentiate into monocytes, macrophages, and neutrophils (myeloid cells), in a process called hematopoiesis. HSCs and GMPs are highly proliferative cells and require LDL-c uptake for their proliferation, as we previously reviewed [29]. Interestingly, JAK2 inhibition with ruxolitinib increases plasma LDL-c levels in animal models and humans carrying the *JAK2* p.Val617Phe mutation [30]. Thus, in a model where JAK2 activation is pronounced, JAK2 activation regulates plasma LDL-c levels.

**FIGURE 1:**
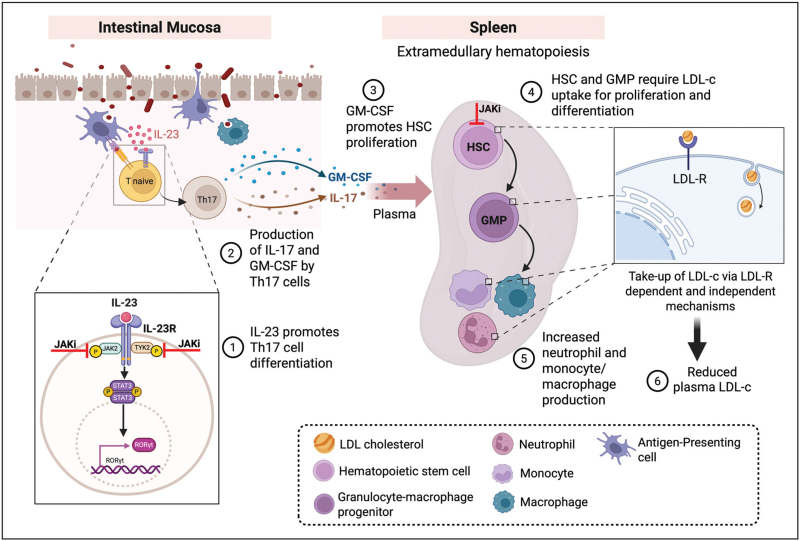
The interleukin-23 (IL-23)–T-helper cell 17 (Th17) axis drives granulocyte–macrophage colony-stimulating factor (GM-CSF)-mediated extramedullary hematopoiesis and low-density lipoprotein-cholesterol (LDL-c) uptake in the context of inflammatory bowel disease (IBD). In the inflamed intestinal mucosa of IBD, antigen-presenting cell (APC)-derived IL-23 promotes Th17 differentiation via JAK/STAT signaling. IL-23 enhances the secretion of GM-CSF by Th17 cells. GM-CSF drives HSC mobilization from the bone marrow to the colon and spleen, and subsequent HSC differentiation towards granulocyte/macrophage progenitors (GMPs), increasing neutrophil and monocyte/macrophage production. In the spleen, HSCs, GMPs, monocytes, and macrophages take up LDL-c, contributing to reduced plasma LDL-c levels. Pharmacological inhibition of the IL-23–Th17 axis by JAK inhibitors (JAKi) restrains Th17 differentiation and downstream GM-CSF production, limiting extramedullary hematopoiesis and reducing LDL-c uptake by progenitors and myeloid cells, increasing plasma LDL-c. JAKi also inhibit HSC self-renewal, as such limiting extramedullary hematopoiesis.

For other JAK isoforms than JAK2, no mutations that clearly affect plasma LDL-c levels have been described. We thus rely on studies with JAK1 or JAK3 inhibitors to investigate a causal relationship between JAK1/3 activity and plasma LDL-c levels, even though mechanisms may share similarities with those reported for JAK2. Along these lines, it has been shown that JAK inhibitors, especially JAK1 inhibitors, may directly decrease HSC self-renewal as such decreasing hematopoiesis [31], which may, conversely to carriers of the *JAK2* p.Val617Phe mutation that show increased hematopoiesis and low LDL-c, result in elevated LDL-c. In addition, other mechanisms may contribute to effects of JAK inhibition on LDL-c in IBD, including the IL-23–T-helper cell 17 (T_h_17) axis. This axis is central to IBD pathogenesis [32^▪^,^33^,^34^]. IL-23 is crucial for the final step of T_h_17-cell differentiation [35]. T_h_17 cells produce IL-17A, and IL-17A plasma levels are elevated in IBD, with reversal by JAK inhibition [33,34,36–38]. IL-23 stimulates granulocyte–macrophage colony stimulating factor (GM-CSF) secretion by T_h_17 cells [35]. In the context of colitis, GM-CSF promotes HSC mobilization from the bone marrow to colon and spleen and HSC proliferation and skewing towards GMP differentiation, not only in the colon, where this aggravates intestinal inflammation and colitis, but also in the spleen [39], in a process termed extramedullary hematopoiesis (i.e. hematopoiesis outside of the bone marrow). It has been shown, as we reviewed previously, that extramedullary hematopoiesis in the spleen, similar to hematopoiesis, decreases plasma LDL-c levels [29]. Conversely, we propose that decreases in IL-23 and IL-17A secretion due to JAK inhibitor therapy suppress T_h_17 production and GM-CSF secretion by T_h_17 cells, consequently decreasing extramedullary hematopoiesis and HSC and GMP proliferation, as such raising LDL-c levels (Fig. 1).

This hypothesis is supported by studies showing a link between GM-CSF therapy and plasma cholesterol levels. A phase I/II clinical trial in patients with aplastic anemia showed that GM-CSF therapy decreased serum total cholesterol levels (by 27–53%), which was reversed by discontinuation of treatment [40]. Similarly, in rabbits, GM-CSF administration reduced plasma total cholesterol, reflected by a decrease in plasma LDL-c [41]. Thus, evidence independent of studies in IBD patients indicates that pathways stimulated by GM-CSF signaling, such as hematopoiesis, are linked with a decrease in plasma LDL-c.

The consequences of JAK inhibition in IBD for cardiovascular risk remain to be addressed. While LDL-c clearly is the main cardiovascular risk factor, an elevated cardiovascular risk in *JAK2* p.Val617Phe mutation carriers who have low plasma LDL-c levels [42,43] suggests that mechanisms increasing leukocyte counts and inflammation predominate in the relationship between JAK activity and cardiovascular risk.

## EFFECTS OF JANUS KINASE INHIBITORS ON THE ENDOTHELIUM IN INFLAMMATORY BOWEL DISEASE

Active IBD is associated with endothelial activation, reflected by increased plasma levels of vascular cell adhesion molecule-1 (VCAM-1), E-selectin, and intercellular adhesion molecule-1 (ICAM-1), which are secreted by endothelial cells and promote MACE by enhancing monocyte adhesion [44,45]. A recent mechanistic study exploring the impact of multiple JAK inhibitors on endothelial cell activation demonstrated that although all tested JAK inhibitors reduced IL-6 production by human umbilical vein endothelial cells (HUVECs) in response to a combined incubation with tumor necrosis factor (TNF) and IL-17A, only three out of six decreased IL-8 production, two increased VCAM-1 expression, and all six increased ICAM-1 expression [46^▪^]. Importantly, these effects were observed at the highest concentration (10 μmol/l), which likely exceeds clinically achieved exposures for JAK inhibitors under standard therapeutic dosing. At 1 μmol/l, a concentration more commonly considered within a clinically relevant range, effects were different: only two JAK inhibitors reduced IL-8 production, one increased VCAM-1, and another one increased ICAM-1. Although these findings suggest that some JAK inhibitors may enhance endothelial activation, this remains to be confirmed by future studies.

## PRO-THROMBOTIC EFFECTS OF JANUS KINASE INHIBITORS AND THEIR RELATIONSHIP WITH VENOUS THROMBOEMBOLISM IN INFLAMMATORY BOWEL DISEASE

The 2019 FDA boxed warning for JAK inhibitors highlighted a potential risk of blood clots, supported by data from the ORAL Surveillance trial, which identified higher rates of venous thromboembolism (VTE) compared with a TNF inhibitor in high-risk RA patients [2]. Although derived outside the IBD population, these findings prompted consideration of thrombotic pathways in the context of JAK inhibition, especially related to VTE.

JAK inhibitors have shown to increase platelet activation and aggregation in animal models and in ex-vivo models, which has been proposed to be downstream of thromboxane A2 production [47] or attenuation of glycoprotein VI (GPVI)-mediated responses [48].

While these preclinical studies suggest that JAK inhibitors modulate platelet function, no clinical data on IBD patients have demonstrated increased bleeding or clinically significant platelet dysfunction with the JAK inhibitors tofacitinib or upadacitinib. Moreover, clinical trials and integrated analyses have not demonstrated a significantly increased incidence of VTE events in patients with IBD treated with JAK inhibitors [49,50]. Although some studies report a numerical trend toward higher VTE rates with higher doses of JAK inhibitors, these observations have not reached statistical significance, and absolute event rates remain low [51]. Future studies are thus needed to determine whether modulation of platelet pathways by JAK inhibitors impacts thrombotic risk in IBD.

## THERAPIES FOR JANUS KINASE-INHIBITOR-ASSOCIATED LDL-CHOLESTEROL CHANGES IN INFLAMMATORY BOWEL DISEASE

With limited IBD outcome data, recent consensus guidance advises managing JAK inhibitor-related lipid changes as part of routine cardiovascular risk care [8^▪▪^], and highlights statins as first option, because they lower LDL-c and decrease cardiovascular risk and mortality [52]. However, translating this general approach to IBD requires careful consideration of an individual risk profile.

IBD patients carry an elevated risk of metabolic syndrome-associated type 2 diabetes [53^▪^] which puts them at increased cardiovascular risk. Statins may enhance the risk of insulin resistance and type 2 diabetes in patients who are prediabetic [54], and may thus not be preferred for the ~20 to 30% of IBD patients at risk of developing metabolic syndrome and type 2 diabetes. These patients may benefit from alternative LDL-c-lowering approaches, although these also may have limitations.

Ezetimibe [55,56] has been associated with gastrointestinal side effects, with diarrhea reported as a common adverse effect in the FDA drug label information [57]. In addition, there is evidence that Proprotein Convertase Subtilisin/Kexin type 9 (PCSK9) inhibition [58] is associated with increased IBD risk, although this association is derived from human genetic analyses [14,59], rather than clinical trials of PCSK9 inhibitors.

Beyond lipid-lowering strategies, minimizing systemic JAK exposure could be expected to limit systemic effects, including LDL-c increases, while preserving mucosal anti-inflammatory efficacy. In this context, gut-selective JAK inhibitors such as izencitinib (TD-1473) have been evaluated in IBD [60]. However, clinical trials with these agents have not yet been successful in inducing remission of ulcerative colitis and Crohn's disease [61], as also shown in the terminated clinical trial RHEA (NCT03758443) [62], presumably because effective JAK inhibition requires adequate intracellular drug levels in immune cells that extend beyond the mucosal surface, which low-absorption strategies may not reliably achieve [63].

Importantly, in the absence of definitive evidence that JAK inhibitor-associated LDL-c increases translate into excess MACE in typical IBD populations, a universal ‘add-on lipid-lowering drug’ approach risks avoidable polypharmacy. Pill burden, side effects, and imperfect long-term adherence are real-world constraints.

We therefore argue for a staged, risk-stratified approach that preserves patient safety without unnecessarily restricting access to effective therapy. Practically, baseline cardiovascular risk assessment (age, weight, personal and family history, blood pressure) and a lipid profile should be obtained before starting therapy, and dietary support should start from day 1 of induction, ideally with dietician-led counselling and follow-up. Repeat lipid profiles 4–8 weeks after starting treatment (end of induction), when LDL-c changes usually appear and often stabilize. Consider lipid-lowering medication only if LDL-c remains clearly elevated on repeat testing (two measurements 4–6 weeks apart during maintenance) and is clinically meaningful given the patient's overall cardiovascular risk. Bile acid sequestrants such as colesevelam or cholestyramine may be considered for LDL-c lowering in IBD patients with elevated LDL-c during JAK inhibition and no obstruction concern [64]. Both agents are licensed for LDL-c reduction, but their value specifically for JAK inhibitor-associated lipid changes in IBD needs prospective study.

## CONCLUSION

Although cardiovascular risk has been raised as a concern for JAK inhibitors, this has not been clearly detected in IBD cohorts. However, the absence of evidence may reflect limited power and treatment channeling, because IBD patients generally do not carry as many cardiovascular risk factors compared to RA patients where the increase in cardiovascular risk as a result of JAK inhibition was originally described [65]. Even so, JAK inhibition is associated with increases in plasma LDL-c levels in some, though not all IBD patients (Table 1). We propose a mechanism linking JAK inhibition to the increase in plasma LDL-c levels (Fig. 1) and suggest that the extent of the increase in LDL-c may be due to dosing, disease severity, and the impact of JAK inhibition on the disease; with a major impact (that affects hematopoiesis) likely leading to increases in LDL-c. Currently, the main question is which LDL-c changes matter clinically in IBD and which mitigation strategies improve outcomes without undermining access to effective therapy.

**Table 1:** Clinical evidence on LDL-cholesterol changes and major adverse cardiovascular events during Janus kinase inhibition therapy in inflammatory bowel disease

Study	JAK inhibitor	Type of study	Participants	Disease	LDL cholesterol (LDL-c)	Major adverse cardiovascular events (MACE)
Isufi *et al.* (2025) [6^▪▪^]	Baricitinib Upadacitinib Tofacitinib Decernotinib	Systematic review and meta-analysis of phase 2 and 3 placebo-controlled randomized clinical trials (RCTs)	3978 patients 1680 controls CD study 183 treated 37 controls	Mainly RA studies (9), and one CD study.	Pooled analysis: LDL-c: increase of 10.66 mg/dl (8.92–12.4, *P* < 0.0001) CD LDL-c: increase of 9.9 mg/dl (4.0–15.7, *P* = 0.0009) at week 16. Higher increase with higher doses: 16.63 mg/dl (8.20–25.05) and 17.01 mg/dl (6.51–27.51) for the 24 and 48 mg dose, respectively	Not available
Danese *et al.* (2022) [16]	Upadacitinib	Phase 3, placebo-controlled RCT consisting of two replicate induction studies and a single maintenance study	996 patients 664: upadacitinib 332: placebo	UC	Induction studies Only LDL-c-related measure: LDL-c:HDL-c ratio which significantly changed in one out of two induction groups: -0.14 (95% CI −0.24 to −0.03). Maintenance study LDL-c:HDL-c ratio: unchanged	No MACE in any treatment group during the induction studies. One MACE (acute myocardial infarction) in the placebo group during the maintenance study
Biemans *et al.* (2020) [20]	Tofacitinib	Prospective cohort study	123 patients	UC	Induction therapy LDL-c: increase by 21.2% (95% CI: 10.5–32%, *P* = 0.001) at week 8.	No thromboembolic events up to week 24 of follow-up. No data on MACE reported
Feagan *et al.* (2021) [18]	Filgotinib	Phase 2b/3 placebo-controlled RCT	1348 patients 1069: filgotinib (100 or 200 mg) 279: placebo	UC	Induction study LDL-c increased by 10 mg/dl and 14 mg/dl for filgotinib 100 and 200 mg groups, respectively (vs. 6 mg/dl with placebo) at week 10 of follow-up. Maintenance study From the maintenance baseline (week 10) to week 58: LDL-c increased by 4– 5 mg/dl	One cardiovascular event in the placebo group (0.4%) during the induction study, and one transient ischemic attack in the filgotinib 100 mg group (0.6%) during the maintenance study
Sandborn *et al.* (2017) [21]	Tofacitinib	RCTs including two induction trials (OCTAVE 1) and OCTAVE 2), and one maintenance trial (OCTAVE Sustain)	Induction studies: 1139 patients 905: tofacitinib 10 mg twice daily 234: placebo Maintenance study: 592 patients 198: tofacitinib 5 mg twice daily 196: tofacitinib 10 mg twice daily 198: placebo	UC	Induction studies LDL-c: increase of 16.3 mg/dl (SD 27.3) and 20.9 mg/dl (SD 30.1) for OCTAVE 1 and OCTAVE 2, respectively at week 8. Maintenance study LDL-c: increase of 7.7 mg/dl (SD 24.2) and 9.8 mg/dl (SD 25.5) at week 52 for tofacitinib 5 and 10 mg groups, respectively	During the induction studies, five cardiovascular events (CVE): three, tofacitinib group; two, placebo group. In the maintenance study, six CVE: five, tofacitinib 5 mg group; one, tofacitinib 10 mg group. No events in the placebo group.
Schreiber *et al.* (2023) [66]	Tofacitinib	Post hoc analysis of Phase 2 (induction study), Phase 3 (induction and maintenance studies), OCTAVE (sustain study), and OCTAVE open (open-label, long-term extension study)	1157 patients (1124 for the assessment of MACE) Patients with prior atherosclerotic cardiovascular disease (ASCVD) *n* = 45 Patients without prior ASCVD *n* = 1112	UC	Only baseline LDL-c levels reported. Lower baseline LDL-c in the group with prior ASCVD compared to patients without prior ASCVD, but a higher percentage of these patients was on lipid-lowering drugs (51.1%) compared to patients without prior ASCVD (29.4%; 20,5%; 7,7%; 2,6% in the high, intermediate, borderline, and low CVD risk groups, respectively)	MACE was low (8/1124 events, 0.7%, IR 0.28) in all patients. Baseline mean 10-year ASCVD risk was higher in patients with MACE than in patients without MACE (13.5 vs. 2.5%, respectively)
Sandborn *et al.* (2023) [67]	Tofacitinib	Long-term safety analysis pooling data from phase 2, phase 3, and open-label extension studies from the global tofacitinib UC clinical development program	1157 patients receiving one or more doses of tofacitinib 5 or 10 mg twice daily. 82.5% of patients received 10 mg twice daily	UC	Baseline LDL-c reported exclusively for patients who presented with MACE. Five of these patients (55.5%) had LDL-c >130 mg/dl. In the Phase 3b/4 study, 90 patients (7.8%) started receiving lipid-lowering therapy, and 22 patients (1.9%) had an increase in the dose of lipid-lowering agents	9/1157 MACE in all studies. MACE was only observed in patients with at least one cardiovascular risk factor (smoking history, obesity, or a previous CVE). Older age and history of diabetes were the only significant predictors of MACE in multivariable analyses
Thomas *et al.* (2025) [12^▪▪^]	Tofacitinib Upadacitinib Filgotinib	Systematic review and meta-analysis	81 703 individuals 79357 IBD patients	CD and UC	Not available	MACE HR: 1.23 (95% CI 1.13–1.34, *I*^2^ = 0.81)
Viola *et al.* (2023) [68]	Tofacitinib	Retrospective cohort study	67 patients Group 1, *n* = 30 (older patients with prior CVD or other comorbidities) Group 2, *n* = 37 (no comorbidities)	UC	Not available, but lipid-lowering drugs (mainly statins) were prescribed during the first weeks of treatment in four patients of each group	No MACE or venous thromboembolism (VTE).
Solitano *et al.* (2025) [69]	Tofacitinib Upadacitinib Baricitinib	Systematic review and meta-analysis of head-to-head comparative effectiveness studies in immune-mediated inflammatory diseases (IMIDs)	80 983 patients treated with JAK inhibitors (JAKi) 280 533 patients treated with anti-TNF IBD studies CD and UC JAKi: 1161 anti-TNF: 28 518	Mainly RA studies (37); two IBD studies had MACE measurements	Not available	No significant difference in MACE between both groups, neither in the pooled analysis nor in the individual IBD studies
Ahuja *et al.* (2025) [49]	Tofacitinib Upadacitinib	Retrospective cohort study	10 278 patients: 856: JAKi (82% UC, 18% CD) 9422: anti-TNF (44% UC, 56% CD)	UC and CD	Not available	No significant differences between both groups in univariate, multiple sensitivity, and stratified analyses. HR: 0.50 (95% CI 0.19–1.30) Only significant difference in high cardiovascular risk patients (>50 years of age, diabetes, hypertension, hyperlipidemia, smoking, history of coronary artery disease), where JAKi-treated patients showed a lower incidence of MACE than those on anti-TNF therapy. HR: 0.10 (95% CI 0.01–0.69)
Yang *et al.* (2025) [70▪]	Tofacitinib Upadacitinib Filgotinib Deucravacitinib Izencitinib Peficitinib Ritlecitinib Brepocitinib Ivarmacitinib	Systematic review and network meta-analysis of phase 2 and 3 RCTs	10 537 IBD patients 7608: JAKi 2929: placebo	UC and CD	Not available	No significant increase in odds ratio (OR) of MACE, VTE, or CVE with any JAKi in the pooled analysis, sensitivity and subgroup analyses
Elgendy *et al.* (2025) [71]	Filgotinib	Systematic review and meta-analysis of RCTs	1681 patients 1138: filgotinib 543: placebo	CD	Not available	No significant difference between filgotinib (100 or 200 mg) and placebo-treated patients in risk ratio (RR) of MACE
Loftus *et al.* (2025) [72]	Upadacitinib	Long-term extension study for the efficacy and safety of upadacitinib.	673 patients 221: upadacitinib 15 mg 229: upadacitinib 30 mg 223: placebo	CD	Not available	No MACE in the long-term study (48 weeks of follow-up) for both groups (upadacitinib 15 and 30 mg), or in the cumulative maintenance population (both groups combined) up to 285 weeks of exposure. 1 VTE in the upadacitinib 30 mg group: 0.3 E/100PY (48 weeks of follow-up), 0.2 E/100PY (up to 285 weeks of exposure).
Panaccione *et al.* (2025) [73]	Upadacitinib	Interim analysis of the U-ACTIVATE long-term extension study	1489 patients 863: upadacitinib 15 mg 626: upadacitinib 30 mg	UC	Not available	Events up to week 96 Upadacitinib 15 mg group: MACE *n* = 6 (0.3/100PY) VTE *n* = 10 (0.5/100PY) Upadacitinib 30 mg group MACE *n* = 3 (0.2/100PY) VTE *n* = 8 (0.5/100PY) 80% of the 15 mg group and 60% of the 30 mg group who presented with MACE had cardiovascular risk factors at baseline
Falcon *et al.* (2025) [74]	Upadacitinib	Systematic review and meta-analysis of RCTs.	2611 patients 1853: upadacitinib 758: placebo	CD and UC	Not available	The incidence of MACE and VTE was too low to perform any statistical comparison. Number of cases VTE *n* = 3 (2 in the upadacitinib group, 1 in the placebo group). MACE *n* = 2 (upadacitinib group)
Colombel *et al.* (2025) [75]	Upadacitinib	Post hoc analysis of the U-EXCEL, U-EXCEED, and U-ENDURE trials	143 patients with perianal fistulizing CD 96: upadacitinib 47: placebo	CD	Not available	No MACE or thromboembolic events at week 52 in upadacitinib or placebo groups
Song *et al.* (2025) [76]	Tofacitinib	Retrospective cohort study	1816 patients 521: tofacitinib 1295: anti-TNF	UC	Not available	MACE, pulmonary thromboembolism (PTE), and deep vein thrombosis (DVT) were comparable between the two groups. IR: 2.74 E/100PY in the tofacitinib group. IR: 3.87 E/100PY in anti-TNF group (*P* = 0.151)

ASCVD, atherosclerotic cardiovascular disease; CVD, cardiovascular disease; CVE, cardiovascular events; DVT, deep vein thrombosis; E/100PY, events per 100 patient-years; HR, hazard ratio; IMIDs, immune-mediated inflammatory diseases; IR, incidence rate; JAKi, Janus kinase inhibitors; MACE, major adverse cardiovascular events; PTE, pulmonary thromboembolism; RCT, randomized clinical trial; VTE, venous thromboembolic event. Target specificity – pan-JAK inhibitors: tofacitinib (preferential selectivity for JAK1 and JAK3), izencitinib, peficitinib; JAK1 inhibitors: upadacitinib, baricitinib (activity also against JAK2), filgotinib, brepocitinib (activity also against TYK2), ivarmacitinib; JAK3 inhibitors: decernotinib, ritlecitinib; TYK2 inhibitors: deucravacitinib.

Future studies should move beyond describing lipid trajectories and directly test whether LDL-c changes under JAK inhibition mediate MACE risk in IBD, and whether risk differs by dose, JAK inhibitor selectivity, and patient phenotype. Linking longitudinal lipid measures to hematopoiesis and myeloid markers, alongside adjudicated cardiovascular and VTE outcomes, will be essential to replace avoidance-based prescribing with evidence-based, efficient risk stratification and mitigation.

## Acknowledgements

*None*.

### Financial support and sponsorship

*K.M. is supported by the Colombian Colfuturo Scholarship program and the UMCG Top-up funding. E.A.M.F. is supported by a ZonMW Clinical Fellowship grant (project number 90719075) and has received a research grant from Takeda. M.W. is supported by the Netherlands Organization of Scientific Research (NWO) VIDI-grant 917.15.350, a NWO Aspasia grant, and a UMCG Rosalind Franklin Fellowship with an EU Co-Fund attached.*

### Conflicts of interest

*There are no conflicts of interest.*

## REFERENCES AND RECOMMENDED READING

Papers of particular interest, published within the annual period of review, have been highlighted as:

▪ of special interest
▪▪ of outstanding interest
